# Body mass index and physical fitness among Chinese adolescents from Shandong Province: a cross-sectional study

**DOI:** 10.1186/s12889-019-6420-2

**Published:** 2019-01-17

**Authors:** Xiangren Yi, You Fu, Ryan D. Burns, Yang Bai, Peng Zhang

**Affiliations:** 1 School of Physical Education, Shandong University, Jinan, Shandong China; 2 School of Community Health Sciences, University of Nevada Reno, Reno, NV USA; 3 Department of Health, Kinesiology, and Recreation, University of Utah, Salt Lake City, UT USA; 4 Department of Rehabilitation and Movement Science, University of Vermont, Burlington, VT USA; 5 Department of Exercise Science, East Stroudsburg University, East Stroudsburg, PA USA

**Keywords:** Body mass index, Children, China, Fitness, Health

## Abstract

**Background:**

This study aimed to describe the most recent prevalence estimates of adolescent body mass index (BMI) and physical fitness from thirteen regions in Shandong Province, China and to examine differences by social-economic status (SES).

**Methods:**

The participants were 27,955 adolescents (mean age = 14.4 ± 1.8 years; 13,791 girls) enrolled from 91 public middle and high schools randomly selected from 13 administrative regions in Shandong Province. The Chinese National Student Physical Fitness Standard was employed to assess participants’ fitness once during the school semester. Fitness assessments included BMI, a 50-m sprint run, sit-and-reach, standing-broad jump, sit-ups, pull-ups, and a 1000 m/boy or 800 m/girl distance run. Participants’ fitness performance was categorized as excellent, good, pass, or no pass.

**Results:**

The percent of Chinese adolescents categorized as “no pass” ranged from just 8.9% for BMI to 67.1% for boy pull-ups. The percent of Chinese adolescents categorized as “excellent” ranged from 5.0% for the girl sit-ups to 35.4% for the 50-m sprint run. Approximately 8.4% of the sample was categorized as excellent for BMI. SES significantly predicted both girl and boy continuous distance run scores (*p* < 0.05). Adolescents in lower SES strata had lower odds of achieving “pass” or better on cardio-respiratory endurance tests, muscular fitness tests, and flexibility tests, but higher odds of achieving “pass” or better on BMI compared to adolescents who were high SES (*p* < 0.05).

**Conclusions:**

The large sample of the adolescents in Shandong province, on average, had healthy weight status and achieved a high prevalence of “pass” or better on physical fitness tests. Adolescents who were low SES demonstrated poorer cardio-respiratory endurance, muscular fitness, and flexibility test achievement but better BMI achievement compared to high SES adolescents in Shandong Province, China.

## Background

Physical fitness is positively associated with youth’s health and academic performance [1, 2]. Previous studies have explored the relationship between physical fitness and health, indicating that physical fitness has a correlation with youth’s cognition, weight status, bone health, and psychological well-being [2–5]. Specifically, cardiorespiratory endurance has been linked with metabolic, cognition and academic achievement [6, 7], muscular fitness is favorably associated with adiposity, insulin sensitivity, bone health, and psychological health of youth [8, 9], and flexibility affects youths’ ability to function and be physically active [10].

Because of the positive impact of physical fitness on an individual’s health, a systematic assessment of physical fitness in youth, such as body composition and cardiorespiratory endurance, provides directions to development and implementation of early health prevention programs. China is facing a challenge in a rising trend of obesity and physical inactivity as the development of the economy and technology [11–15]. Surveillance data shows a rapid growing level of sedentary behaviors along with a rising trend in obesity among Chinese youth and childhood overweight and obesity, which no longer unusual in the country [11–15]. Specifically, large scaled research indicated a prevalence of overweight and obese young increased from 5.5 to 26.3% in last fifteen years [16–18]. An inverse association was found between children’s body composition and cardiorespiratory fitness and muscular explosive strength based on an investigation of six thousand children aged from 6 to 12 years old [17]. These data highlight that promoting an active and health healthy lifestyle among Chinese school student remains a critical challenge in practice.

Previous studies have indicated the relationship between obesity and physical fitness performance in Caucasian children [15, 19]. Even though ethnic differences in body composition are evident with a higher percentage of body fat mass and less lean mass in Asian children than other ethnicity groups at the same BMI level [19–22], limited study was conducted in the relationship among the physical fitness domains through a large sample in Chinese children. Because there are more than 116 million children attending schools in China, the need for monitoring and tracking school students’ physical fitness remains a high physical education and public health priority. Fitness testing in China consists of the annual physical fitness test through school physical education time mandated by the Ministry of Education in China. Specifically, physical fitness is assessed by employing using the Chinese National Student Physical Fitness Standard (CNSPFS) battery [19]. These tests have demonstrated reliability and validity evidence in Chinese youth [19]. There has been regular public health surveillance in China in order to track and monitor various fitness levels such as body composition, muscular strength, and cardio-respiratory endurance [1]. However, recent fitness test prevalence estimates from adolescents from Shangdong Providence have not been reported and there may be significant variation in BMI and fitness test scores by socio-economic status (SES) because of the relatively large discordance in SES in this specific area in the People’s Republic of China. Therefore, the purpose of the study was to present the most recent prevalence estimates of BMI and physical fitness in Chinese school aged adolescents in Shangdong Province and to examine differences in those outcome measures by sex and SES strata. The updated data and the knowledge gained through this effort are likely to inform the development of health promotion policies and programs for Chinese youth.

## Methods

### Participants and study design

**Fig. 1:**
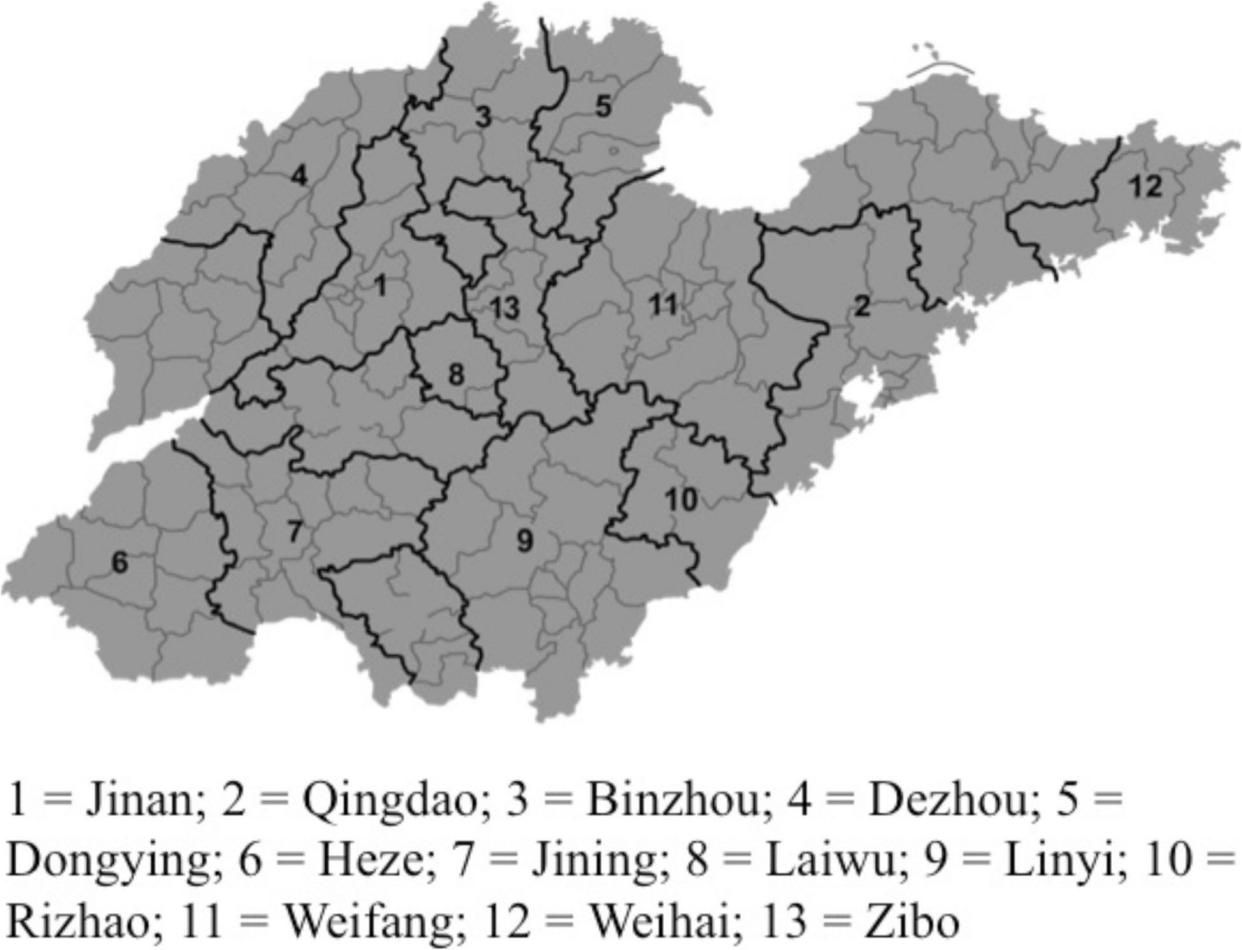
Illustration of the thirteen selected sampling regions of Shandong Province, China

The present cross-sectional study was conducted during the 2016–2017 academic year in Shandong Province, China. A 3-stage cluster sampling method to recruit a regionally representative sample of adolescents from 91 public middle and high schools from 13 administrative regions including Binzhou, Dongying, Dezhou, Heze, Jining, Jinan, Laiwu, Linyi, Qingdao, Rizhao, Weifang, Weihai, and Zibo (see Fig. 1). These 13 regions were randomly selected to ensure the geographical diversity of the samples.

A total of 28,062 secondary school students aged 12–19 years (14.5 ± 4.3 years) participated in the fitness assessments and 27,955 participants (14,164 boys and 13,791 girls) were eventually included in the analysis after removal of extreme scores (107/28,062; < 1% of total sample). A total of 102 evaluators were recruited from physical education (PE) teachers working in middle and high schools who had previous experience in evaluating youth fitness and who had operated National Student Fitness Test program, which aims to promote physical activity for school children and youth in China. All evaluators were given a testing manual that had been developed by the project team and illustrated all the test guidelines, procedures and protocols. In addition, all evaluators completed two training seminars that facilitated to standardize and homogenize the method of assessment and quality control in order to reduce intra- and inter-tester errors. All elevators organized students to assess physical fitness test and guidance to answer electronic-based questionnaires.

### Assessments

Trained physical education (PE) teachers administered all tests by following standard operating procedures. The training was administered through workshops. Participants completed a standardized form, which included socio-demographic data, region, school, age, gender, date of birth, grade and ethnicity. Data collection was incorporated with the annual physical fitness test through the school physical education time mandated by the Ministry of Education in China. Physical fitness was assessed by employing using the Chinese National Student Physical Fitness Standard (CNSPFS) battery [19], which contains seven tests that gauged different components of fitness. Each fitness test score was calculated by a grade- and sex-specific percentage, then categorized into “not pass”, “pass”, “good”, and “excellent”. The testing battery is a reliable and valid instrument to assess physical fitness in adolescents and is a norm testing battery in China [19]. The test-retest reliability across all assessments employed in the current study were ICC > 0.90, which was determined acceptable.

#### Body mass index (BMI)

BMI was selected as a surrogate assessment of body composition. Participants’ height (cm) was measured to the nearest 0.1 cm in bare feet while weight (kg) were examined to the nearest 0.1 kg by GMCS-IV; Jianmin, Beijing, China). Based on the height and weight results, BMI scores were calculated as weight in kilograms divided by squared height in meters (kg/m^2^). Participants from all the grade years recruited in the study (7th–12th grade) received the BMI assessment

#### 50-m Sprint run

This sprint test was administered on a flat and clear surface where participants were instructed to run in a straight line for 50 m (m). Each participant performed the test one time as a single maximum sprint and the performance was recorded to the nearest 0.1 s. All participants were required to complete the test

#### Standing long jump

Participants stood behind a starting line marked on the floor with two feet together. The participants then jumped forward with maximum power and the results were measured in distance from the take off line to the nearest point of contact on the landing (back of the heels). Three attempts were allowed and the longest distance (in cm) was recorded as the official score

#### Sit-and-reach

The sit-and-reach test reflects flexibility level of the lower body. Participants were instructed to take a seated position with both knees fully extended and feet placed firmly against a vertical support. They were requested to reach forward with their hands as far as possible along a measuring line. Each participant performed the sit-and-reach test for two trails with the score on the farthest distance recorded (measured to the nearest 0.1 cm)

#### 1000 M/800 m distance run

A sex-specific test of participants’ cardiorespiratory endurance, which instructed a 1000-m run and an 800-m run for boys and girls, respectively. Participants were instructed to run as fast as possible for the distance requested while being allowed to walk or stop during the test. Running performance was recorded to the nearest 0.1 s

#### Timed sit-ups (girls)

Timed sit-ups was selected as an assessment of abdominal muscle endurance. The test instructed participants to perform sit-ups as many times as possible for one minute. The testing staff counted the number of sit-ups during the period. The standard of a qualified sit-up was described as “to lay in a supine position with the knees bent and feet flat on the floor mat with their hands placed on the back of the head and fingers crossed”. The participants elevated their trunk until their elbows made a contact with the thighs. The participants then returned to the starting position by lowering their shoulder blades to the mat. The final score was recorded as the number of successfully completed repetitions

#### Pull-ups (boys)

Pull-ups were used to indicate upper body muscular endurance. Assuming an upright position, with a long jump, children grasped an overhead bar using an overhand grip with arms fully extended. Children were asked to use their arms to pull the body up until the chin cleared to the top of the bar and them lower their body again to a position with the arms extended. The final score was recorded as the number of successfully completed repetitions

### Statistical analysis

Data were checked for Gaussian distributions using k-density plots. Extreme outliers were removed from the data set using a z-score cut-point of ±5.0. Differences between the sexes on all observed variables were examined using independent t-tests. Effect sizes were calculated using Cohen’s delta (d), where d < 0.20 indicating a small effect, d ≈ 0.50 a medium effect, and d ≥ 0.80 a large effect [23]. To examine the independent predictive relationships between each fitness test continuous score and SES, multi-level general linear mixed effect models were employed. Random intercepts were used at the region level. Likelihood ratio tests with chi-square statistics were employed to test if the multi-level models were statistically different from the naïve model assuming no clustering within the data structure. Analyses were conducted for the total sample and within sex groups to test for sex modifying effects. Age was entered in as a covariate within each of the models. The reporting of the results included the adjusted parameter (b-coefficient) estimates with 95% Confidence Intervals.

To examine the predictive relationship between categorical fitness test achievement (i.e., no pass, pass, good, and excellent) and SES status, multi-level ordered logistic models using STATA’s “meologit” command were employed. A likelihood ratio test and the Brant test were employed to test the proportional odds assumption of ordered logistic regression, which assumes that the computed coefficients that describe the relationships among all levels of the dependent variable (i.e., levels of achievement) are invariant. Reporting of the results included the age-adjusted odds ratios (ORs) with corresponding 95% Confidence Intervals. The reference level for fitness test achievement was the “no pass” category. Categorical analyses were run for the total sample and within sex groups to test for modifying effects. All analyses had an a priori alpha level set at *p* ≤ 0.05 and were carried out using STATA v15.0 statistical software package (College Station, Texas, USA).

## Results

The descriptive statistics are presented in Table 1. Boys recorded higher BMI (mean difference = 0.6 kg / m^2^, *p* < 0.001, d = 0.17), shorter 50-m sprint run times (mean difference = − 1.0 s, *p* < 0.001, d = 0.77), a longer standing long jump distance (mean difference = 28.9 cm, *p* < 0.001, d = 0.65), and shorter sit-and-reach scores compared to girls (mean difference = − 3.4 cm, *p* < 0.001, d = 0.44). There were no mean age differences between the sexes. Mean fitness test scores within specific Regions are communicated in Table 2. Across Regions, mean BMI ranged from 19.0 to 20.0 kg/m^2^, mean sprint run scores ranged from 7.8 to 9.3 s, mean long jump scores ranged from 165.4 to 191.6 cm, mean sit-and-reach ranged from 8.1 to 33.6 cm, mean girl distance run ranged from 224.6 to 268.7 s, mean boy distance run ranged from 256.4 to 298.0 s, girl’s sit-ups ranged from 26.8 to 37.0 repetitions, and boy’s pull-ups ranged from 3.9 to 6.0 repetitions.

**Table 1:** Descriptive statistics for the total sample and within sex groups (means and standard deviations)

	Total Sample (*N* = 27,955)	Girls (*n* = 13,791)	Boys (*n* = 14,164)
Age (years)	14.4 (1.8)	14.4 (1.8)	14.3 (1.8)
BMI (kg/m^2^)	20.2 (3.6)	19.9 (3.3)	**20.5 (4.0)**
Sprint Run (sec)	8.7 (1.3)	**9.2 (1.2)**	8.2 (1.2)
Long Jump (cm)	180.5 (43.6)	165.6 (31.3)	**194.5 (48.7)**
Sit-and-Reach (cm)	11.6 (7.6)	**13.3 (7.0)**	9.9 (7.7)
Girl Distance Run (sec)	–	247.9 (40.9)	–
Boy Distance Run (sec)	–	–	272.7 (53.5)
Sit-up (Girls; reps)	–	31.8 (10.6)	–
Pull-up (Boys; reps)	–	–	5.1 (5.5)

BMI stands for Body Mass Index; bold denotes statistical significance, *p* ≤ 0.05

**Table 2:** Descriptive statistics by Region (means and standard deviations)

	BMI (kg/m^2^)	Sprint run (sec)	Long jump (cm)	Sit-and-Reach (cm)	Distance Run (Girl) (sec)	Distance Run (Boy) (sec)	Sit-ups (Girls)	Pull-ups (boys)
Binzhou	20.3 (3.3)	8.5 (1.0)	189.1 (30.4)	9.1 (7.1)	239.8 (29.8)	266.3 (38.2)	32.6 (8.0)	3.9 (4.2)
Dongying	19.0 (3.2)	8.74 (1.3)	175.3 (42.8)	12.0 (5.8)	224.6 (28.0)	260.8 (43.8)	28.3 (11.4)	6.0 (7.6)
Dezhou	20.0 (3.5)	9.3 (1.1)	179.5 (32.5)	33.6 (2.4)	247.6 (29.8)	278.4 (57.0)	28.3 (8.7)	4.0 (3.7)
Heze	20.0 (3.1)	8.7 (1.3)	165.4 (65.5)	12.0 (7.6)	254.1 (40.0)	274.9 (55.0)	27.1 (11.7)	5.8 (5.8)
Jining	20.1 (3.7)	8.9 (1.3)	181.0 (38.4)	8.1 (7.1)	268.7 (44.9)	298 (56.0)	29.9 (10.2)	4.4 (4.4)
Jinan	20.7 (3.6)	7.8 (0.8)	188.9 (35.3)	17.9 (3.5)	256.7 (44.5)	273.8 (58.4)	32.5 (9.9)	4.7 (5.4)
Laiwau	20.5 (3.8)	8.9 (1.2)	177.3 (46.7)	11.3 (6.8)	246.7 (39.1)	278.7 (52.5)	35.0 (8.6)	3.6 (4.0)
Linyi	20.0 (3.4)	8.6 (1.2)	191.6 (35.0)	13.1 (6.3)	244.7 (41.5)	266.0 (51.5)	26.8 (10.7)	7.1 (5.8)
Qingdao	20.5 (3.7)	8.4 (1.2)	187.0 (29.9)	11.5 (6.7)	242.2 (31.5)	264.1 (43.5)	31.6 (10.5)	4.5 (4.0)
Rizhao	20.1 (3.5)	8.5 (1.1)	184.9 (34.6)	9.7 (7.9)	252.8 935.4)	273.7 (45.2)	30.0 (9.9)	6.0 (5.8)
Weifang	19.9 (3.8)	8.8 (1.6)	170.0 (49.3)	12.1 (7.3)	249.6 (40.0)	271.8 (51.4)	31.5 (10.4)	5.3 (6.1)
Weihai	20.9 (4.3)	8.6 (1.1)	177.4 (56.4)	10.8 (6.7)	234.8 (44.4)	256.4 (61.2)	36.6 (8.9)	4.9 (5.9)
Zibo	20.6 (3.9)	8.7 (1.1)	185.6 (31.6)	14.1 (6.6)	232.5 (35.6)	262.8 (42.7)	37.0 (10.0)	5.4 (6.7)

The results of the general linear models are presented in Table 3. SES significantly predicted distance run scores for both girls and boys, respectively (*p* < 0.05). Interestingly, the lower SES strata tended to take longer times to complete the 800 m and 1000 m distance run tests (signifying lower cardio-respiratory endurance) compared to children belonging to the highest SES strata > 100,000 RMB. There were no other relationships found on any of the other continuous fitness test variables.

**Table 3:** Fixed-effect parameter estimates from general linear mixed effects models

Fitness Test	SES Strata (gross domestic product per capita)	Total Sample b-coefficient (95% C.I.)	Girls b-coefficient (95% C.I.)	Boys b-coefficient (95% C.I.)
BMI (kg/m^2^)	50,000–99,999 RMB	0.30 (−0.27, 0.89)	0.40 (−0.12, 0.91)	0.23 (−0.46, 0.93)
	0–49,999 RMB	−0.02 (−0.65, 0.60)	0.03(−0.52, 0.58)	− 0.07 (− 0.82, 0.68)
Dash Run (sec)	50,000–99,999 RMB	− 0.11 (− 0.47, 0.25)	− 0.17 (− 0.65, 0.31)	−0.06 (− 0.48, 0.35)
	0–49,999 RMB	0.25 (− 0.17, 0.60)	0.16 (− 0.35, 0.68)	0.28 (− 0.16, 0.73)
Long Jump (cm)	50,000–99,999 RMB	5.0 (−6.9, 17.0)	−2.3 (−12.7, 8.1)	2.1 (−4.0, 28.1)
	0–49,999 RMB	1.3 (−11.5, 14.2)	−5.4 (−16.6, 5.8)	7.4 (−9.9, 24.7)
Sit and Reach (cm)	50,000–99,999 RMB	0.22 (−9.2, 9.7)	0.16 (−8.53, 8.9)	0.24 (−9.9, 10.4)
	0–49,999 RMB	5.1 (−5.1, 15.3)	4.4 (−4.5, 13.8)	5.9 (− 5.1, 16.9)
Girl Distance Run (sec)	50,000–99,999 RMB		18.5† (4.6, 32.5)	
	0–49,999 RMB		**26.8†** **(11.8, 41.8)**	
Boy Distance Run (sec)	50,000–99,999 RMB			15.8† (2.2, 29.5)
Sit-up (Girls; reps)	50,000–99,999 RMB		−0.24 (−4.8, 4.3)	
	0–49,999 RMB		−2.7 (−7.6, 2.2)	
Pull-ups (Boys; reps)	50,000–99,999 RMB			−1.05 (−2.5, 0.4)
	0–49,999 RMB			−0.39 (−2.0, 1.1)

b-coefficients are adjusted for clustering within Region and age; 95% C.I. stands for the 95% Confidence Interval; RMB stands for Renminbi; BMI stands for Body Mass Index; SES stands for Socio-Economic Status; referent for SES is ≥100,000 RMB; bold and † denotes statistical significance, *p* ≤ 0.05

**Fig. 2:**
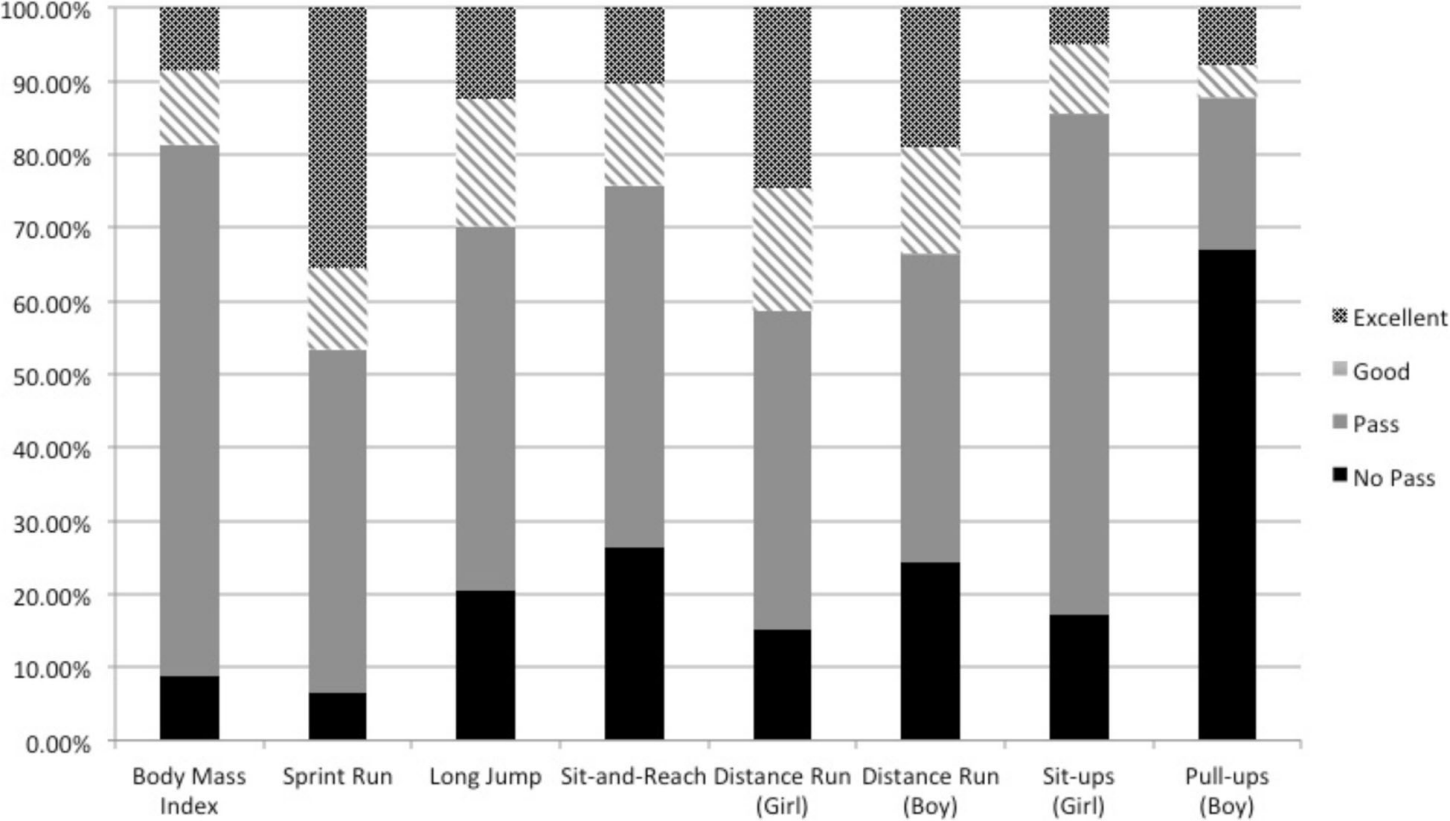
Fitness test standard achievement

Figure 2 presents the fitness test standard achievement for the total sample. The percent of Chinese adolescents categorized as “no pass” ranged from just 8.9% for BMI to 67.1% for boy pull-ups. The percent of Chinese adolescents categorized as “excellent” ranged from 5.0% for the girl sit-ups to 35.4% for the 50 m sprint run. Approximately 8.4% of the sample was categorized as excellent for BMI. The results of the multi-level ordered logistic regression models are reported in Table 4. For BMI, adolescents in the lower SES strata had higher odds of achieving “pass” or better compared to adolescents who were high SES > 100,000 RMB. However, for all of the other fitness assessments including the sprint run, long jump, sit-and-reach, girl and boy distance runs, sit-ups, and pull-ups, lower SES adolescents had significantly lower odds of achieving at least “pass” or better compared to higher SES adolescents (*p* < 0.05). Sex only modified standing long jump scores, as significant relationships were found in girls but not in boys. For all linear and non-linear models, the likelihood ratio tests were significant, confirming the use of multi-level modeling and suggesting significant variation among the sampling regions.

**Table 4:** Fixed-effect parameter estimates from the multi-level ordered logistic models

Fitness Test	SES Strata	Total Sample OR (95% C.I.)	Girls OR (95% C.I.)	Boys OR (95% C.I.)
BMI (kg/m^2^)	50,000–	**1.41†**	**1.39†**	**1.51†**
	99,999 RMB	**(1.29, 1.53)**	**(1.22, 1.58)**	**(1.31, 1.75)**
	0–49,999	**1.42†**	**1.38†**	**1.55†**
	RMB	**(1.30, 1.55)**	**(1.21, 1.57)**	**(1.34, 1.80)**
Dash Run (sec)	50,000–	**0.79†**	**0.68†**	0.91
	99,999 RMB	**(0.70, 0.88)**	**(0.60, 0.73)**	(0.82, 1.02)
	0–49,999	**0.63†**	**0.58†**	**0.71†**
	RMB	**(0.55, 0.72)**	**(0.52, 0.66)**	**(0.63, 0.79)**
Long Jump (cm)	50,000–	**0.70†**	**0.71†**	1.00
	99,999 RMB	**(0.66, 0.76)**	**(0.65, 0.79)**	(0.91, 1.11)
	0–49,999	**0.95†**	**0.76†**	1.01
	RMB	**(0.86, 0.99)**	**(0.69, 0.84)**	(0.91, 1.11)
Sit-and-Reach (cm)	50,000–	**0.51†**	**1.49†**	**0.63†**
	99,999 RMB	**(0.47, 0.55)**	**(1.34, 1.65)**	**(0.57, 0.70)**
	0–49,999	**0.62†**	**0.59†**	**0.71†**
	RMB	**(0.58, 0.68)**	**(0.53, 0.65)**	**(0.64, 0.78)**
Girl Distance Run (sec)	50,000–		**18.5†**	
	99,999 RMB		**(4.6, 32.5)**	
	0–49,999		**26.8†**	
	RMB		**(11.8, 41.8)**	
Boy Distance Run (sec)	50,000–			**0.52†**
	99,999 RMB			**(0.47, 0.57)**
	0–49,999			**0.54†**
	RMB			**(0.49, 0.60)**
Sit-ups (Girls; reps)	50,000–		**0.72†**	
	99,999 RMB		**(0.64, 0.81)**	
	0–49,999		**0.51†**	
	RMB		**(0.46, 0.58)**	
Pull-ups (Boys; reps)	50,000–			1.03
	99,999 RMB			(0.92, 1.15)
	0–49,999			**1.15†**
	RMB			**(1.03, 1.29)**

OR stands for Odds Ratio that is adjusted for clustering within Region and age; 95% C.I. stands for the 95% Confidence Interval; RMB stands for Renminbi; BMI stands for Body Mass Index; SES stands for Socio-Economic Status; referent for SES is ≥100,000 RMB; bold and † denotes statistical significance, *p* ≤ 0.05

## Discussion

The purpose of the study was to characterize the most up-to-date physical fitness prevalence of Chinese school-aged adolescents in Shangdong Province and to examine the differences in BMI and physical fitness by sex and SES. In general, results of the present study indicated that the large sample of Chinese adolescents had healthy weight status, in that the mean BMI was in the “normal” zone based on the Chinese National Student Physical Fitness Standard [19] and that less than 9% of the sample fell within the “no pass” category. The results also suggested that the Shandong Province adolescents in the present study demonstrated a relatively good physical fitness level, with the lone exception being boy pull-ups, where more than 67% fell within the “no pass” category. The scores of the fitness tests were above the “pass” standard [12] for most of the other fitness assessments. Finally, BMI and physical fitness varied by SES, especially for the girl and boy distance runs and for most assessments when analyzing the data by achievement categories. Thus, SES may be an important determinant of body composition and physical fitness in adolescents from Shangdong Province.

Among the total sample of 27,955 secondary school students in the present study, the average BMI (20.2 kg/m^2^) was higher compared to the BMI (17.3 kg/m^2^) another recent study conducted in a large-scale (*n* = 171,991) Chinese youth [19]. However, in the current study, approximately 8.9% of the participants’ BMI were in the “no pass” zone, and a majority (> 70%) of adolescents in Shandong province demonstrates a “pass” BMI level but approximately only 8.4% of the participants’ BMI was categorized as “excellent”. Regarding sex differences, boys had higher BMI (mean difference = 0.6 kg / m^2^) than girls.

In terms of the fitness levels, adolescents in Shandong Province generally demonstrated good physical fitness status. The average percentage of adolescents within the “pass” categories for the sprint run, long jump, sits & reach, distance run, and sit-ups (girls). However, nearly 70% of the participants failed to pass the pull-ups (boy) standard, indicating a concern on boy’s upper body strength. Nevertheless, there was a low prevalence of “excellent” performance on the physical fitness tests. For instance, the “excellent” category on sit & reach, sit-ups, and pull-ups was less than 10%. Less than 20% of the participants could achieve “excellent” in long jump and boy’s distance run. Regarding the sex difference in physical fitness, boys generally demonstrated more an optimal physical fitness level than girls, in that boys achieved significant better performance on the dash run and standing long jump distance. The overall low prevalence of “excellent” on the physical fitness may be partially due to the sedentary lifestyle among the adolescents reported by the recent studies [20, 21], and this trend may eventually lead to the greater risk of overweight and obesity [22], and other chronic diseases.

The results in the present study were echoed by previous research. For example, a study reported a decline among American children and adolescents’ physical fitness during the past 20 years [24]. The study on 2010 National Physical Fitness and Health Surveillance also suggested a low level of physical activity, with about 77% of primary and junior middle school children in China could not meet the national recommendations [21]. The 2016 Physical Activity and Fitness in China-The Youth Study (PAFCTYS) also reported additional evidence, which indicated that the Chinese youth’s overall physical fitness level was relatively low, with about 10% of the youth not meeting the national recommendations from the descriptive statistics [18].

Social-economic status (SES) may explain the BMI and physical fitness regional differences. Based on the 2016 and 2017 Shandong Statistical Yearbook released by the Shandong Provincial Bureau of Statistics, the gross domestic product per capita of Dongying and Qingdao were ranked the 1st and 3rd regions respectively, in Shandong Province, as compared to Dezhou, Laiwu, Jining, whose gross domestic product per capita ranked 11th–13th places [25, 26]. In general, adolescents in the low and middle SES strata had poorer achievement on most fitness tests compared to high SES children. Paradoxically, this was the opposite for BMI, where lower SES children had higher odds of achieving “pass” or better compared to high SES children. Poorer BMI achievement in high SES can be explained by increased food supply and possibly a positive energy balance compared to lower SES children. However, it is also possible that higher BMI may be due to increased fat-free mass and not fat mass, as higher SES children tended to perform better on most fitness tests, requiring high levels of muscular strength and endurance.

The relationship between SES and fitness is supported by other theoretical perspectives. Gesell [27] claimed that children and adolescents’ social environment plays a significant role in gross motor skills development, which in turn, affects physical activity participation and physical fitness levels. Stodden et al. [28] suggested that young individuals’ experiences influence various levels of physical activity level and physical fitness. These experiences include the environment, physical education, SES, and parental support, etc. Additionally, Venetsanou and Kambas [29] also reported that family SES influenced children’s physical fitness development. In the review, Venetsanou and Kambas [29] found that in a majority of relevant studies children of lower SES tend to perform worse than those of the middle/high SES in fitness performance. A number of posssile reasons may explain why the lower SES adolescents in the present study may have poorer motor competence. Low SES adolescents’ lower physical fitness level may also be associated with poor nutritional habits, which may affect muscular development. Additionally, lower SES adolescents may not be encouraged to develop physical fitness during school day, as they do not have regularly scheduled physical education classes. In out-of-school settings, low SES adolescents living in disadvantaged communities may suffer from the lack of facilities that preclude optimal development of physical fitness [30–32]. Furthermore, higher SES adolescents may greater resources compared to lower SES adolescents.

There are limitations to this study that must be considered before the results can be generalized. First, the sample was recruited from Shandong province in the Eastern Region of China and the overwhelming majority of the sample was of Han ethnicity; therefore, the external validity of the results is questionable if generalized to adolescents located in other regions and to populations with different ethnic distributions. Second, body composition was assessed using BMI and the other fitness tests were assessed using field assessments. The construct validity of the assessed domains would be stronger if lab-setting measurements were employed especially for body composition (e.g., dual energy x-ray absorptiometry, hydrostatic weighing) and cardio-respiratory endurance (measured VO_2 Peak_). Additionally, physical fitness was assessed using the Chinese National Student Physical Fitness Standard, which was customized based on the Chinese students’ characteristics. Therefore, the implication of the results should be re-considered if generalized to adolescents in other countries. Finally, study variables were assessed once due to logistical reasons; therefore, causal relationships cannot be made because of the cross-sectional research design, thus attenuating the internal validity.

## Conclusions

In conclusion, the large sample of the Chinese adolescents in the present study had healthy weight status with less than 9% in the “no pass” category. Additionally, a large prevalence of Chinese adolescents achieved the physical fitness “pass” standard or better on most fitness tests; however there seems to be a large area of improvement because of the low prevalence of adolescents within the “excellent” category. The only assessment where the majority of the sample did not meet the “pass” criterion was for boy pull-ups, suggesting a need for significant improvement for boy upper body muscular strength. Finally, adolescents in lower SES regions demonstrated lower physical fitness achievement on most assessments compared to those from high SES regions in Shandong Province, China. There remains room for improvement in the overall fitness levels among Chinese adolescents, and thus it is imperative to continue surveillance and develop interventions and program in and out of school aimed at promoting physical activity participation and fitness, especially in lower SES regions and remote areas. According to each test, cut-points were used to classify physical fitness in the four excellent categories. With this information, other professionals who work with this population will be able to classify the physical fitness of the adolescent population with ease.

## Abbreviations

BMI: Stands for body mass index
PE: Stands for physical education
SES: Stands for social-economic status
